# Recent Developments in Printing Flexible and Wearable Sensing Electronics for Healthcare Applications

**DOI:** 10.3390/s19051230

**Published:** 2019-03-11

**Authors:** Saleem Khan, Shawkat Ali, Amine Bermak

**Affiliations:** College of Science and Engineering, Hamad Bin Khalifa University, Qatar Foundation, Doha 5825, Qatar; shaali@hbku.edu.qa (S.A.); abermak@hbku.edu.qa (A.B.)

**Keywords:** wearable electronics, biosensors, nanomaterials, printed electronics, flexible substrates

## Abstract

Wearable biosensors attract significant interest for their capabilities in real-time monitoring of wearers’ health status, as well as the surrounding environment. Sensor patches are embedded onto the human epidermis accompanied by data readout and signal conditioning circuits with wireless communication modules for transmitting data to the computing devices. Wearable sensors designed for recognition of various biomarkers in human epidermis fluids, such as glucose, lactate, pH, cholesterol, etc., as well as physiological indicators, i.e., pulse rate, temperature, breath rate, respiration, alcohol, activity monitoring, etc., have potential applications both in medical diagnostics and fitness monitoring. The rapid developments in solution-based nanomaterials offered a promising perspective to the field of wearable sensors by enabling their cost-efficient manufacturing through printing on a wide range of flexible polymeric substrates. This review highlights the latest key developments made in the field of wearable sensors involving advanced nanomaterials, manufacturing processes, substrates, sensor type, sensing mechanism, and readout circuits, and ends with challenges in the future scope of the field. Sensors are categorized as biological and fluidic, mounted directly on the human body, or physiological, integrated onto wearable substrates/gadgets separately for monitoring of human-body-related analytes, as well as external stimuli. Special focus is given to printable materials and sensors, which are key enablers for wearable electronics.

## 1. Introduction

**Figure 1:**
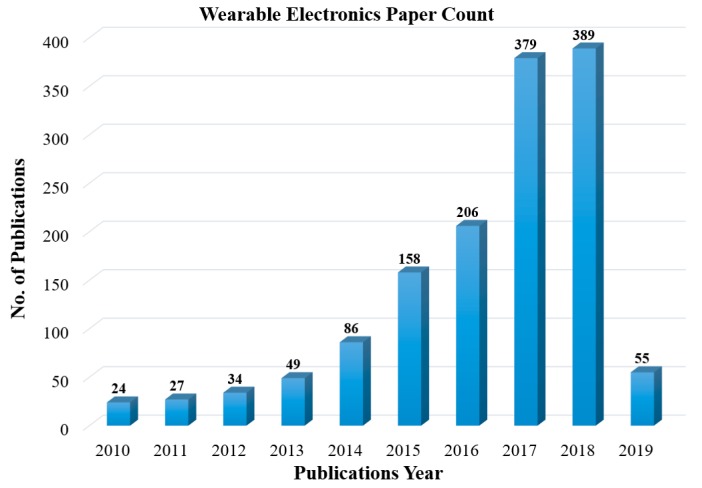
Number of publications per year with titles including wearable electronics (Source: NCBI (National Center for Biotechnology Information)).

**Figure 2:**
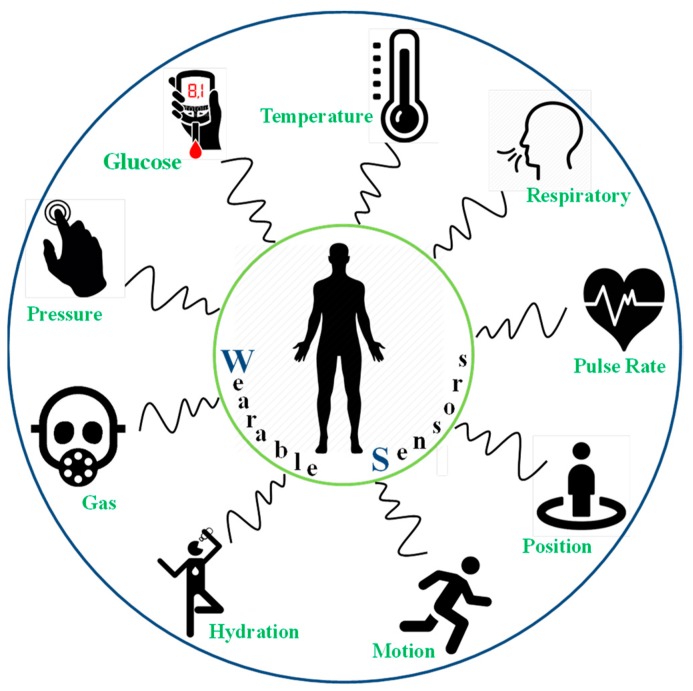
Schematic of representative wearable healthcare sensing devices.

The development of sensors and electronics on unconventional, non-planar, conformable substrates through cost-effective manufacturing routes diversified the application areas [1,2,3,4]. The rapid emergence of a wide range of nanomaterials with enhanced sensitivities (compared to their bulk counterparts) and solution processability offered a promising outlook for these sensors to be developed and operated at room temperatures [5,6]. Among the latest developments, wearable sensors and electronics attracted significant interest for their capabilities to monitor human health status in real time [5,7,8,9,10]. Figure 1 shows the trend of publications in recent years targeting wearable electronics applications. A wide variety of chemical, physical, and optical sensors are embedded separately or combined onto flexible substrates with complementary data readout and signal conditioning circuits [11,12,13,14,15]. Data are transmitted wirelessly to nearby computing devices or in the cloud, and are examined by medical experts who give respective commands as per the health conditions [5,16,17]. Polymeric substrates are ideal for employing these sensing devices and circuits for their burgeoning properties such as their light weight, low cost, flexibility, bendability, foldability, stretchability, and conformability to uneven surfaces with negligible losses to the sensor data [18,19,20]. In the current scenario of the development of wearable electronics, biosensors related to in situ monitoring of human biological fluids, physiological activities, and gaseous analytes in the surrounding environment that directly affect human health are of particular interest [21,22,23,24]. A few representative examples are mentioned in the schematic of Figure 2. Developing wearable sensors for healthcare-related applications faces a multitude of challenges, including the selection of suitable substrates, biocompatible materials, and manufacturing techniques, as well as the simultaneous monitoring of different analytes, the washability, uninterrupted signal readout circuits, etc. The recent developments of all-organic biocompatible or hybrid sensors on wearable substrates are paving the way to realizing body-worn sensing systems for in vivo monitoring.

The development of wearable sensors on polymer substrates requires a compatible manufacturing process that respects the chemical and thermal properties of the substrates, as well as enabling cost-effective fabrication on large areas. Printing technologies are the most promising manufacturing approaches for such developments that deposit solution-based functional materials at desired locations with minimal processing steps [25,26,27,28]. The print-on-demand approach makes these technologies attractive due to the effective utilization of materials in the development process. A wide range of materials are deposited on diverse substrates, covering larger areas as compared to the conventional Si (silicon) substrates [26,29,30]. The lower material cost, less manufacturing waste, and low-cost fabrication techniques, among others, are the key attractions of printing technologies [26,31]. Printable inks are commonly nanoparticle dispersions in suitable solvents, and rheological properties are tuned according to the processing requirements of the respective printing technology [32]. Nanoscale materials are ideal for sensing applications due to their higher surface-to-volume ratio and are, therefore, applied for different sensing applications [25,28,33]. The printing of electronic components on flexible substrates widened the application areas of sensory systems, especially wearable biosensors which are applied for in vivo monitoring of biological fluids, as well as physiological activities, in real time. In this context, printed wearable electronics gained great momentum, whereby new strategies are adopted to conformably integrate sensing patches directly on the human body or in the form of wearable gadgets for various human health-related biomarkers. This review covers the latest major developments, particularly in the last five years, and encompasses the advancements made in various aspects, such as materials, substrates, manufacturing, sensor types, sensing mechanism, readout circuits, and wireless data transmission. A brief overview of the challenges in developing and adopting wearable electronic technology and the future scope of the field are also summarized in the final section of this article.

## 2. Wearable Sensors and Electronics

**Figure 3:**
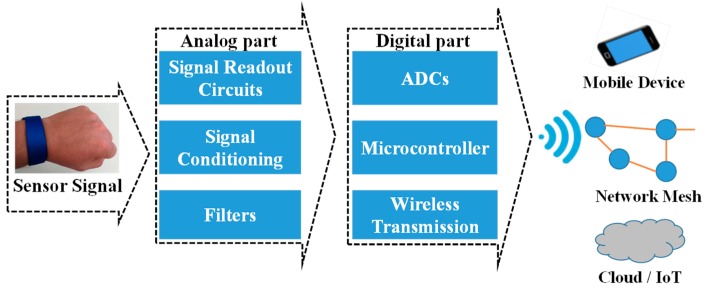
Signal flow diagram for measuring an entity through wearable sensors and data transmission.

Advances in wearable sensors and electronics witnessed significant interest and adoptability, particularly in the areas of health fitness monitoring, entertainment, the fashion industry, etc. [1,4,34]. The focal research interest lies in developing biosensors that can easily be integrated onto wearable substrates/gadgets for continuous health monitoring [35]. Wearable sensors are foreseen to improve the medical care system, particularly for the elderly and patients with chronic diseases that require continuous monitoring/supervision. The schematic in Figure 3 provides an overview of the sensing mechanism, whereby raw data generated by a sensor connected to the human body are processed and transmitted to medical experts remotely by connecting wearable systems through a wireless transmission system. Most of these sensors are based on the monitoring of biological fluids, particularly sweat, which can selectively detect glucose, lactate, cholesterol, and pH levels, among others [14,36,37]. Sweat sensors can further be functionalized to detect various biomolecules and salt concentration levels [9]. The monitoring of human physiological activities such as pulse rate, hydration/dehydration, temperature, motion, pressure, strain, etc. is of particular interest [4,15,38,39]. Human breath analysis is also a good source of biomarker detection as it can be used to monitor breathing rate, deep body temperature, alcohol detection for diabetes, and for a range of volatile organic compounds exhaled in the breath [40,41,42]. The majority of wearable biosensors are developed on a single patch that is capable of simultaneously monitoring these different biomarkers without any considerable cross-talk. The sensor patches are directly integrated onto the human epidermis using biocompatible materials and substrates, or by placing the sensors in textile or other secondary conformable substrates used as part of a wearable gadget [23,43,44,45,46]. The sensing and interconnections are mostly made of solution-based functional materials that can easily be patterned in a very cost-effective manufacturing process, i.e., printing. Sensors are connected to the data readout and signal conditioning circuits, allowing the data to be eventually transmitted to the computing hub or a data analysis expert through wireless communication tools. Handheld mobile phones are the preferred computing tools for the monitoring of individual subjects in most cases, whereas data are sent to clouds when the data of many users have to be analyzed, where an expert opinion is generated and transmitted back to the users. Currently, interest is developing in the heterogeneous integration of printed sensors on polymer substrates, while off-the-shelf electronics are used for faster data processing and communication. The research of the former is attractive and recent developments in nanomaterials presented very promising results for printing multifunctional sensors on similar substrates.

## 3. Printing Technologies

**Figure 4:**
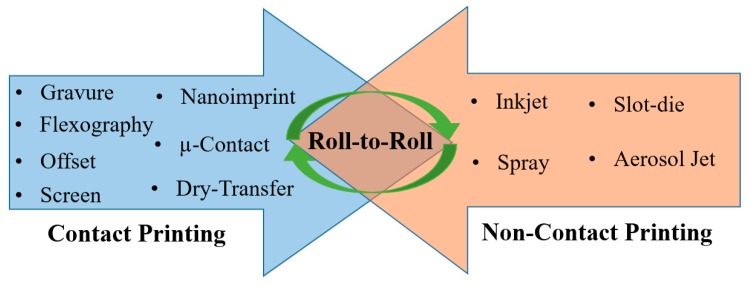
Summary of representative contact- and non-contact-based printing technologies.

Printing is a rapidly growing field for the fabrication of sensors and electronics on non-planar substrates. It involves position-specific deposition of functional materials from their colloidal or chemical solutions. The number of fabrication steps is far less than that for standard microfabrication technology practiced in clean-room processes [26,27,31]. Printing is a bottom-up manufacturing approach, whereby materials are added layer by layer in succeeding fabrication steps. This additive manufacturing makes printing a distinguished process for its simple and cost-efficient approach compared to conventional microfabrication techniques. Printing techniques are broadly divided into two categories (Figure 4) based on contact and non-contact of the printing medium with the target substrates [26]. In the contact-based approach, the printing medium with designed structures on the surface is inked and brought in physical contact with the target substrate. Such techniques involve screen printing, gravure printing, flexographic printing, pad-printing, stamp-assisted transfer printing, etc. [26,47]. Details of these processes are provided elsewhere [26]. In non-contact-based printing, materials are ejected in the form of micro-droplets or a continuous jet, facilitated by miniaturized printing nozzle heads. This is often referred to as digital manufacturing, where droplets/jets are ejected on demand as a result of the respective actuation mechanism [29]. Non-contact printing processes predominantly include piezoelectric inkjet printing, electrohydrodynamic (EHD) inkjet printing, slot-die and aerosol-jet printing, etc. [26,48]. Non-contact approaches are more attractive as they are versatile and allow rapid changes in the designed structures using computer-controlled software [31]. Details of non-contact printing technologies are provided elsewhere [26]. Roll-to-roll (R2R) manufacturing is the ultimate goal of developing such printing technologies. R2R serves as a common platform by installing different printing and in-line curing/sintering systems for the batch manufacturing of electronic components at higher speeds. Nonetheless, each of these printing technologies is central to the development of all- or semi-printed sensing devices and systems.

## 4. Substrates Enabling Wearable Sensors

Substrates play a very significant role in shaping the sensors’ physical, mechanical, and electrical features [20]. The degree of bendability, foldability, and stretchability determines the conformable integration of substrates into non-planar surfaces, which is the core requirement of wearable electronic systems. Thin polymeric sheets with minimum thicknesses are ideal for wearable applications. Polymeric materials, such as polyimide (PI), polyurethane (PU), polyethylene terephthalate (PET), polyethylene naphthalene (PEN), polydimethylsiloxane (PDMS), etc., are some of the representative substrates that are vastly used [18,26]. Their chemical inertness, as well as their thermal and electrical insulation, makes these polymeric substrates ideal for sensor and electronic development [19]. Biocompatibility is one of the major requirements for on-body integrated or epidermal sensors and substrates, with PDMS-, PU-, polylactic acid (PLA)-, and cellulose-based polymer substrates being explored recently [18,19,49]. Additionally, some other non-conventional substrates based on textiles are also used for wearable electronic applications [50].

## 5. Wearable Sensors

**Figure 5:**
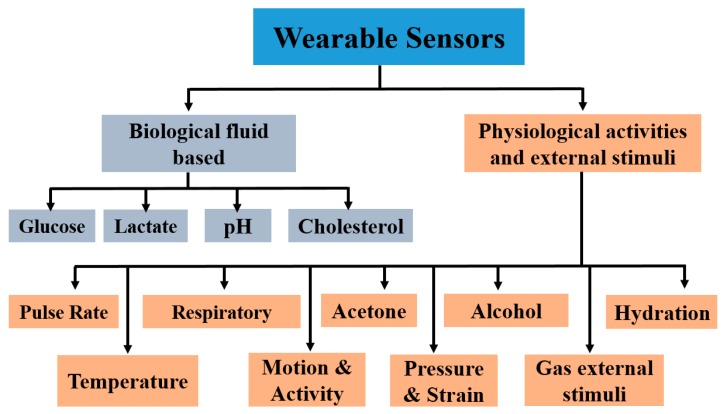
Block diagram of representative and prominent wearable sensors covered in this review article.

This section includes biosensors developed fully or partially using printing technologies. In this review, we divide wearable sensors into two broad categories. The first one is based on the detection of biomarkers present in biological fluid such as sweat, whereby the sensors are attached directly onto the human epidermis. The second one involves sensors based on physiological activities and their monitoring with respective wearable sensors. Figure 5 shows a block diagram of representative and prominent wearable sensor types covered in this review. The geometry, materials, fabrication techniques, and sensing mechanisms are briefly described in each section.

### 5.1. Biological Fluid-Based Sensors

Sweat sensing is a broadly investigated area in wearable electronic applications, where the concentrations of different analytes are assessed concerning human health risks [9,39]. Sweat is a bodily fluid excreted under certain circumstances, and contains several potential analytes related to human health status, such as sodium, chloride, potassium, carbonates, ammonia, calcium, glucose, lactate etc. [39,51,52]. Certain levels of these substances found in the blood, saliva, tears, and sweat are the main bio-recognition sources for assessing human health conditions. Among these, sweat-based sensors are ideal for wearable applications due to the easy and non-invasive deployment onto the epidermis and the simple replacement procedure using disposable sensor patches. The majority of developed wearable sensors are functionalized with specific enzymes to enhance the selectivity and sensitivity toward a specific analyte. Various biosensors were developed for wearable applications and the most prominent ones include glucose sensors, pH sensors, lactate sensors, cholesterol sensors, etc. These sensors are discussed separately in the upcoming sections.

#### 5.1.1. Glucose Sensors

**Figure 6:**
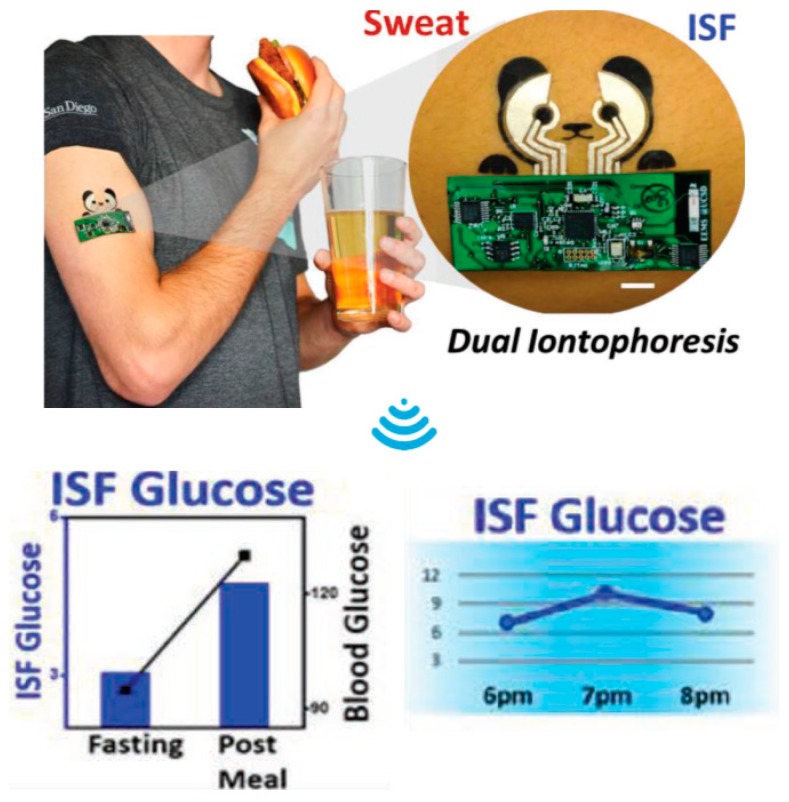
Depiction of a wearable iontophoretic biosensor device on a printed tattoo platform for glucose sensing on a human subject, along with wireless real-time transmission of the interstitial fluid (ISF) glucose [58] (reproduced with permission).

Diabetes is considered as a widespread disease, causing severe health implications around the globe. Millions of people are affected with this disease and it is among the leading causes of death annually as per World Health Organization (WHO) reports [53,54]. Disorder or depletion of insulin production in the human body is the main reason for this disease, causing irregularities in sugar levels. Frequent monitoring of the glucose level is crucial to avoid serious repercussions. Glucose biosensors passed through many developmental stages since their first generation presented in 1962 by Clark and Lyons at the Children’s Hospital in Cincinnati [55]. Different human physiological fluids such as blood, urine, sweat, saliva, interstitial fluid, ocular fluid, and breath contain glucose biomarkers for the detection for diabetes [14,56]. The most prominent approach for selective detection is through enzymatic methods, i.e., glucose oxidase (GOx), whereby redox reactions occur at the interfacing medium [14]. The sensor measures the decrease in oxygen concentration and the liberation of hydrogen peroxide (H_2_O_2_), which is directly proportional to glucose concentration. Starting from lab-level tests by taking blood samples, this technology then emerged into hand-held devices for rapid detection. Recently developed glucometers use disposable enzyme-activated electrode strips [14]. A detailed overview of glucose sensors and their latest development in portable devices is provided elsewhere [14,35,56,57]. With the fast advancement of microelectronics on non-conventional substrates, especially wearable and conformable ones, glucose sensors attracted significant research interest lately [58]. In vivo measurements, as shown in Figure 6, enabled by wearable and implantable glucose sensors, are attractive, as they help the continuous real-time monitoring of patients for quick self-assessment [45]. New approaches, i.e., Internet of things, enable medical experts to monitor and advise patients remotely through the design of cloud-based systems [59]. Several proof-of-concept models were proposed recently and were tested for real-time glucose monitoring.

An all-printed tattoo-based glucose sensor for noninvasive glycemic monitoring was presented in Reference [60]. The device works on the principle of epidermal diagnosis through a combination of reverse iontophoretic extraction of interstitial glucose and enzyme-based amperometric biosensing (Figure 6). Screen printing was used to develop the sensors, using silver (Ag) and silver chloride (AgCl) for the reference and counter electrodes, respectively, on a papilio transfer base paper substrate. Conductive carbon-based ink was used to print the working electrode. A comparison of the measurement results obtained for in vitro and on-body glucose sensing showed promising results for the detection of micromolar levels of glucose. An innovative work recently presented wearable sensor arrays for multiplexed in situ perspiration analysis [61]. A fully integrated wearable system was developed for simultaneous measurements of sweat metabolites and electrolytes. Sensor arrays were developed for the selective detection of glucose, lactate, and sodium and potassium ions, embedded along with sensors for skin temperature measurement. Electrochemical sensors were developed using Ag/AgCl electrodes for glucose and lactate sensing, whereas ion-selective electrodes were activated by drop-casting ion-selective membranes for the precise detection of different electrolytes, i.e., Na^+^, K^+^, etc. The successful implementation of the proposed system for detailed sweat analysis during indoor and outdoor activities of human subjects showed its capability of personalized diagnostic and real-time human health monitoring. Functional materials at a nanoscale level, i.e., nano-wires/ribbons/tubes, play a significant role in the development of conformable sensing devices, ideally for wearable applications. For instance, In_2_O_3_ (indium oxide) nano-ribbons were used to develop field-effect transistor (FET) sensors on PDMS (polydimethylsiloxane) substrates [24]. Sensors were laminated onto human body parts, and different body fluids such as sweat, tears, and saliva were tested for glucose detection down to 10 nM concentrations. A sweat-based glucose sensor integrated with a feedback transdermal drug delivery system was also proposed for efficient measurements [52]. Sensor efficiency was enhanced and made accurate through the real-time correction of pH, temperature, and humidity monitoring. Miniaturized devices were developed through clean-room processes and were ultimately transfer-printed onto a PDMS substrate, which is ideal for conformable and wearable applications. Other than on-body sensors, cotton fabric was also reported for wearable lactate-sensing applications [13]. The electrochemical sensors consisted of carbon graphite, Ag, and AgCl electrodes, printed on cotton fabric as the working, reference, and counter electrodes, respectively. Spray-printing of reduced graphene oxide (rGO) was used as the working electrode in another development of a glucose-sensing device [21]. The sensor was attached to a human wrist, and reported an excellent amperometric response to in vivo glucose detection in the range of 0–2.4 mM. A non-enzymatic robust wearable sensor patch with all the complementary signal processing and communication tools was proposed on flexible stainless steel [45]. Sensors were implanted into sub-cutaneous tissue, and they continuously measured the interstitial fluid glucose at various intervals. Organic material-based biosensors are interesting for wearables, as they comply with the basic requirements of processing at ambient temperature, as well as biocompatibility. A PEDOT(poly(3,4-ethylenedioxythiophene))–glucose oxidase cross-linked biosensor was used for amperometric-based sensing [59]. A three-electrode structure was developed on PET substrate with front-end electronics for the remote monitoring of glucose levels. All these rapid developments in sensing materials and manufacturing processes show a very promising future, and these devices are foreseen to begin industrial production in the near future.

#### 5.1.2. Lactate Sensors

Lactate is one of the key metabolites in the human body which is produced in muscles due to the anaerobic metabolism of glucose. Continuous monitoring of lactate is essential during exercise, particularly for athletes, to avoid cell acidosis, which results in disruption of muscular performance [62,63]. Different fluids in the human body contain a certain level of lactate concentration. For instance, blood contains 0.5–1.5 mM lactate for a healthy person at rest, whereas about 12 mM is present during exercise [64]. However, the level is different in other body fluids such as tears, saliva, sweat, etc. [64,65,66]. Monitoring through wearable sensors is required to be non-invasive, ideally through the analysis of sweat or interstitial body fluids [27,36]. Selectivity is important as the body fluids contain several other metabolites; therefore, enzymes are used in most wearable electrochemical sensors. Enzymes such as lactate oxidase are usually used for selective detection; however, non-enzymatic sensors were also produced [67]. Mediator solutions are sometimes used as a catalyst to enhance the redox reactions.

**Figure 7:**
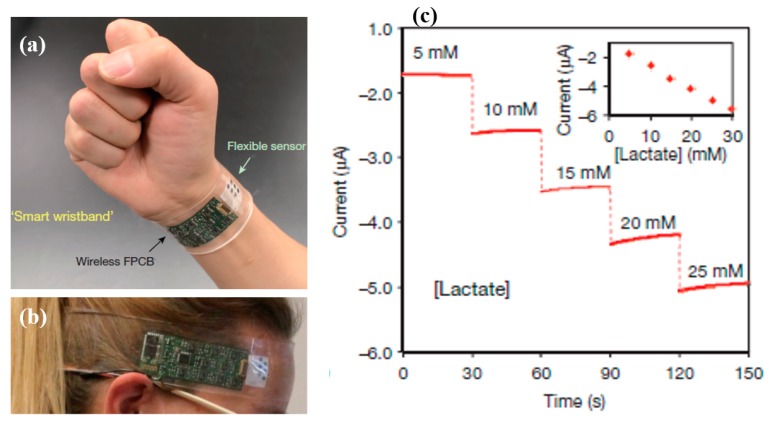
(**a**) Photograph of a wearable sensor array on a subject’s wrist; (**b**) smart headband; (**c**) chronoamperometric responses of lactate at different concentrations [61] (reproduced with permission).

An electrochemical tattoo-based biosensor was developed through printing and was embedded onto human skin for real-time lactate monitoring during exercise [63]. The sensor was developed using Ag/AgCl and functionalized MWCTs (multi-wall carbon nanotubes) in a three-electrode architecture. The sensor showed chemical selectivity toward lactate with linearity up to 20 mM and was more resilient during continuous mechanical deformations. A multiplexed sensor array for simultaneous measurements of multiple metabolites, electrolytes, and skin temperature was developed on a mechanically flexible substrate [61]. The sensor patch was fully integrated and wearable on a human wrist and headband, as shown in Figure 7. A combination of biochemical and electrophysiological sensing patches is a more advanced approach to hybrid sensing devices [68]. Screen printing was used to print Ag/AgCl for electrodes of the sensors, with lactate oxidase as the enzyme. The sensor patch was mounted on the skin and successful on-body epidermal tests were reported for simultaneous measurements of electrophysiological and on-body chemical sensing without any cross-talk in the generated signals. A non-enzymatic sensor was developed using screen printing, and the working surface was electropolymerized with 3-aminophenylboronic acid (3-APBA) with imprinting of lactate [67]. The detection range of lactate was from 3 mM to 100 mM with a detection limit of 1.5 mM at a response time of 2–3 min. Textile-based printed amperometric biosensors were reported for the first time on cotton fabric for lactate detection [13]. Carbon graphite and Ag/AgCl were printed on fabric as working, reference, and counter electrodes, respectively. The sensor was immobilized with lactate oxidase and showed detection levels of 0.05–1.5 mM with a measuring time of five minutes. A tube-based painted biosensor for lactate detection showed promising results [36], representing a portable and accessible contained shape suitable for biochemical analysis. The interior of tube was printed with carbon graphite and Ag/AgCl to construct an electrochemical biosensor. Graphene nano-wells were printed, along with Ag/AgCl, to develop an electrochemical biosensor for real-time measurements of lactate levels [69]. The sensors were tested in different fluidic mediums such as deionized water and phosphate buffer solutions to mimic human body fluids, showing a large index range of 1.0 µM to 10 mM. Lactate sensing is a central focus in the research of wearable electronics and the fast developments both in materials and enabling manufacturing technologies witnessed in recent years show a positive trend toward its acceptability as a commercial product in the near future.

#### 5.1.3. pH Sensors

The pH is the measure of acidity and alkalinity, or the caustic and basic components present in a target solution. Measuring pH is critical and fundamental to various environmental, biological, and chemical processes. Different kinds of detection and measurement methods were developed [70]. For instance, potentiometric, chemiresistive, optical, mass, and capacitive techniques, among others, were conventionally applied to measure the pH levels of corresponding solutions. Conventionally, pH measurement is performed using glass electrodes and ion-selective field-effect transistors (ISFET). The rigidity, requirement of a reference electrode, and the risk of leakage of electrolyte make it more challenging for miniaturization and wearability of the sensors on uneven surfaces [71]. Therefore, new strategies, such as chemiresistive sensing, were considered as the most suitable approach toward low-cost and miniaturized devices, allowing wearability with a minimal effect on the responses. The pH sensing of different analytes in human physiological solutions, especially sweat, attracted significant interest. The pH of patients with type II diabetes and having kidney stones is lower than that of normal human beings [72]. Similarly, several issues related to skin disorders are also dependent on pH values of the subjects. Therefore, skin-mounted, noninvasive, in vivo, and real-time continuous monitoring of pH is foreseen to play a significant role in a timely diagnosis of developing health issues [9].

**Figure 8:**
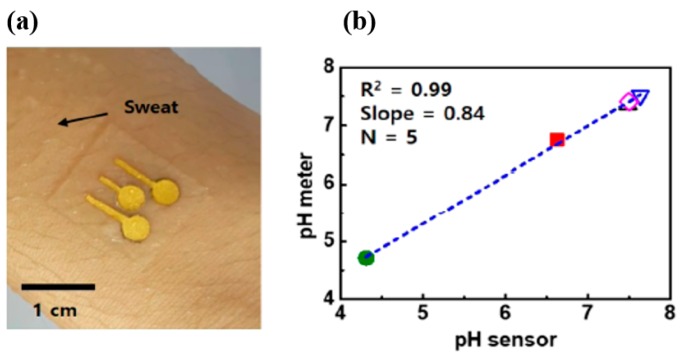
(**a**) Optical image of electrochemical sensor attached to the skin wet with sweat; (**b**) correlation of glucose concentration (left) and pH (right) measured by sensors (*x*-axis) with those measured by a commercial glucose assay kit and commercial pH meter (*y*-axis) in human sweat [9] (reproduced with permission).

The human skin responds to pH changes of the body and can be exploited for continuous pH monitoring as shown in Figure 8. For instance, normal skin is more acidic when hydrated, whereas dehydrated skin tends to be slightly basic in nature. Therefore, research is more focused on utilizing these data with suitable sensors that can distinguish between hydrated and dehydrated conditions. An electrochemical device coupled with data acquisition and signal conditioning circuits was developed for continuous and real-time monitoring of pH and calcium content in human body fluids [72]. The sensing results were validated through spectrometry techniques, as well as through the use of a commercial pH meter, showing high repeatability and selectivity toward target analytes. A capsule-sized implantable pH sensor prototype was presented for gastroesophageal reflux monitoring in the human body [43]. Interdigital electrodes were designed for impedance and pH sensors, which operated wirelessly powered by external transponders. Conductive wire-based electrodes made of cotton yarn were applied for pH and other chemical analytes in the human body [73]. Carbon nanotubes (CNTs) were used as the conductive filler and were coated with a polymeric membrane to develop ion-selective electrodes. Functionalization is key to applying CNT-based sensing devices. Bio-functionalized and inkjet-printed CNTs were used for pH sensing [74]. The doping and de-doping of CNTs by hydronium and hydroxide ions was exploited for pH sensing. Multiple printing cycles enhanced the conductivity with reproducible sensitivity results and a faster response time. Screen-printed thick films of Ag/AgCl/KCl electrodes were presented for the pH and temperature sensing of different solutions [75]. Printed metal-oxide-based (i.e., TiO_2_) thick films were also reported for pH measurements, as well as water quality analysis [70]. Screen printing was adopted to print the IDEs (inter digital electrodes) and a TiO_2_ thick sensing layer. Impedance measurements carried out on test solutions with different pH showed a strong dependency on the pH values. In a recent development, high-resolution aerosol-jet printing was used to pattern CNT-based serpentine-shape sensing layer Ag electrodes [71]. The miniaturized sensor showed good sensing with minimum response time. Being biocompatible, the sensor was claimed to be ideal for live-cell applications. The large number of research outcomes related to simple manufacturing processes on flexible substrates, material development, and accurate detection mechanisms for pH in real time show a positive trend in the adoptability of these devices. Table 1 summarizes the sensing type, materials, substrates, and sensing mechanisms for fluidic-based sensing systems.

#### 5.1.4. Cholesterol

Cholesterol monitoring is vital for human health to have better control over increasing risks to the human body. For instance, an increased level of cholesterol in blood may lead to heart diseases, stroke, high blood pressure, coronary artery diseases, arteriosclerosis, cerebral thrombosis, etc. [76]. Therefore, highly sensitive biosensors for cholesterol monitoring based on different mechanisms were extensively explored. Details highlighting the recent approaches and developments for ameliorating the selectivity and sensitivity of enzymatic-based cholesterol sensors are provided elsewhere [77]. OECT (organic electrochemical transistor)-based sensors are developed for cholesterol monitoring [78]. PEDOT–PSS (polystyrene sulfonate) was incorporated as the channel material in OECT, while functionalizing the gate electrode with cholesterol oxidase and the biocompatible polymer Nafion, to enhance the selectivity toward cholesterol detection. An array of integrated field-effect transistors (i-FETs) was reported for the simultaneous and selective detection of cholesterol, glucose, and urea using multiple analytes [76]. ZnO nanorods were used for making arrays of biosensors highly reliable for the rapid detection of multiple analytes. Very few researches explored the production of sensing devices on polymeric substrates through printing technologies. Wearable cholesterol sensors are in their infancy and need special interest from the research community, especially from the point of view of materials and detection mechanisms.

### 5.2. Physiological Sensors

#### 5.2.1. Pulse Rate

**Figure 9:**
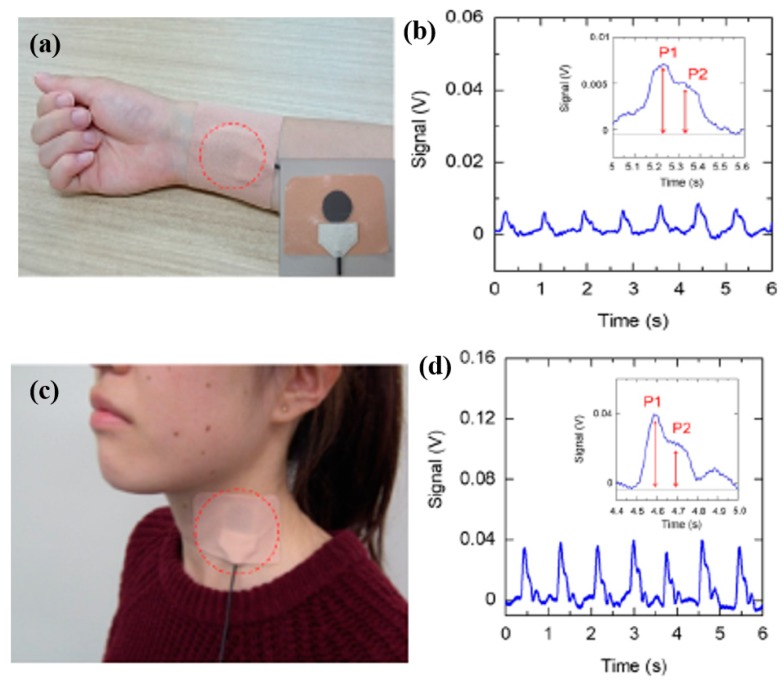
(**a**) Photograph of the as-prepared multifunctional wearable device mounted on the human wrist for simultaneous monitoring of VOC (volatile organic compounds)-related disease and pulse signal; (**b**) normalized resistance changes of the PGF-2 sensor for monitoring wrist pulse and the respiratory rate of a 22-year-old healthy male; (**c**) photograph of a vital sensor attached to the skin of a volunteer using a skin-compatible adhesive patch on the neck; (**d**) real-time monitoring of the pulse [23] (reproduced with permission).

Measuring pulse rate is an interesting biomarker toward real-time human health monitoring, and it can be measured through different approaches. For instance, the difference in the intensity of transmitted and received light from a small light-emitting diode (LED) passing through a part of the human body (especially the finger or earlobe) is conventionally practiced [79,80]. In another approach, pulse rate is measured using highly sensitive pressure sensors mounted on the blood vessels on top of the human skin [80,81]. These two popular techniques were implemented for real-time continuous monitoring of pulse rate by employing different methods of packaging. The LED-based sensing technique matured and can be implemented as standalone or as an integral part of electronic gadgets such as smart watches and wristbands. The bulkiness of the entire package and the development on rigid substrates make it challenging for the sensor to be attached conformably onto the human skin. Therefore, developing sensors on polymeric substrates was actively pursued recently and research is geared toward developing sensors for human-skin attachability [81]. The conformal integration and the biocompatibility of most polymeric substrates are ideal in this scenario, providing a better solution toward wearable and implantable sensing devices. Figure 9 shows representative examples of wearable pulse-rate sensors mounted on the wrist and on the neck of a human subject.

The recent developments in engineered nanomaterials and their solution processability through printing technologies enabled the manufacturing of pressure-sensitive sensors on flexible substrates. One such approach proposed by developing an all-organic optoelectronic pulse sensor on polymer substrate [82] was presented to overcome the issues of bulkiness of conventional pulse-monitoring sensors. Organic LEDs and organic photodiodes (OPDs) were printed on polymeric substrate, which resulted in a high sensitivity with 1% error. Advanced nanomaterials such as PbS quantum dots and blends of PbS with multiwall carbon nanotubes (MWCNTs) were also reported for developing photodetectors on flexible substrates [83]. These sensors were developed on a PET substrate and are suitable for wearable applications to measure the heart rate both in the red and near-infrared ranges. A combination of three sensors (i.e., skin temperature, skin conductance, and pulse rate) was presented to detect human stress level [84]. A multilayer structure was developed, which profoundly reduced the overall assembly size. The pulse wave sensor was developed using a flexible piezoelectric membrane on top of a perforated polyimide substrate. The sensors were claimed to be highly responsive and are central to the wearable multimodal physiological and emotional monitoring of human beings. A multilayered structure was developed through the three-dimensional (3D) integration of multiple components, resulting in a flexible and conformal sensing package for hand gesture and pulse-monitoring applications [85]. The package was assembled using laser processing and soft elastomeric transfer printing approaches. A fully printed pulse-rate monitoring sensor based on a ferroelectric (PVDF–TrFE (polyvinylidene fluoride-co-trifluoroethylene)) polymer was very recently developed [23]. The sensor is sufficiently flexible and conforms to the human skin, providing high pressure sensitivity, a fast response time, and mechanically robust properties.

Some other modules developed by assembling off-the-shelf components were also proposed for an accurate measurement of the pulse rate [86]. A highly flexible, stretchable, and ultrathin wearable microtubular sensor was proposed for pulse-rate monitoring [87]. The sensor was composed of a liquid-state conductive material in a tubular assembly, which was sensitive to minor mechanical perturbations caused by the human arteries. The pressure variance of the surface of the ear canal was also reported for heart-rate monitoring. A piezoelectric film-based sensor was used for pulse monitoring in a scissor-shaped apparatus [88]. A highly flexible, wearable, and disposable biosensor was presented in the latest development for pulse-rate monitoring [89]. All electronic components, including the sensors and wireless communication modules, were sandwiched in thin polyurethane and polyimide substrates with a size similar to that of an adhesive bandage. Functionalized graphene films were used for developing multifunctional wearable sensing devices for the simultaneous monitoring of physiological signals [22]. The systems were capable of monitoring of different volatile organic compounds along with pulse-rate signals.

#### 5.2.2. Respiration

Respiration monitoring sensors are rapidly expanding and are often reported side by side with other iconic human physiological monitoring sensors [90]. Respiration is the breathing rate of a person in a specific period of time. The normal range of respiration rate for a healthy person in normal conditions is 15–20 breaths per minute, whereas values higher than 25 and lower than 12 are considered alarming [91,92]. The respiration rate changes as a result of different physiological perturbations such as asthma, chronic obstructive pulmonary disease (COPD), chronic bronchitis, pneumonia, nasal and sinus blockage, cough, mild fever, etc. Therefore, continuous monitoring of the respiratory rate is of prime importance for the early detection and diagnosis of any occurring irregularities. A variety of sensors were developed for analyzing the respiration rate. For instance, highly precise and ultrasensitive temperature sensors were used to detect nasal activity during breathing [93]. The slight change in temperature due to inhaling and exhaling is considered as a reference for the breathing rate. Polymer-based (PVDF) nasal sensors were also used as cantilevers to exploit the piezoelectric properties upon bending [94]. Such systems are bulky, interruptive, and unfriendly, especially in the case of elderly people. Therefore, wearable technology is highly desirable, as it can be placed on other body parts without interrupting the nasal activity. In this scenario, the most suitable place (i.e., the human chest) is reported to be ideal for these sensors. The chest expansion and contraction upon inhaling and exhaling, respectively, can easily be monitored using strain sensors. 

**Figure 10:**
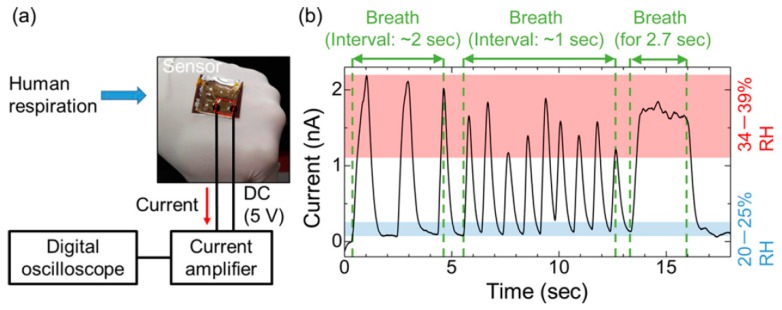
(**a**) Measurement system for monitoring human respiration. The device is mounted on a 125-µm polyimide film for stable measurement. (**b**) Monitoring human respiration using the sensor. The used sensor is indicated by the dashed square in (a) [100] (reproduced with permission).

Large-area printed electronics enabled such a development with much ease, allowing the effective integration onto non-planar substrates. Screen-printed strain sensors based on MWCNT pastes were reported on a textile substrate for breathing-rate measurements [95]. The sensor was embedded into garments, which allowed ease of wearing. The change in electrical resistance as a result of chest expansion and contraction was recorded for each breath in and out. MWCNT/PDMS nanocomposites were also explored for respiratory-rate monitoring using strain sensing based on capacitive structures of inter digitated electrodes (IDEs) [96]. In another development, an innovative approach was proposed for measuring the respiration rate based on humidity sensors printed on paper [97]. The printing of carbon black on both sides of the paper substrate was utilized to produce sensors in the shape of IDEs. The changes in ionic conductivity of the sensor were caused by the changing levels of humidity occurring as a result of breathing. The lightweight sensors were embedded in a wearable mask and the data were transmitted wirelessly to a smartphone. A PVDF-based sensor was proposed for the detection of respiration rate in dynamic walking conditions [98]. A PVDF layer was sandwiched between two printed Ag layers, which generated an electrical signal due to the deformation caused in the human chest. Graphene was investigated recently for a wide range of sensing applications. An all-painted respiration sensor was produced recently using a film of silica nanoparticles combined with a sensitive graphite layer [38]. The sensors were produced on a flexible substrate to enable conformal integration onto a non-planar surface of human body. Graphene-oxide-based sensors were produced in another interesting development, as shown in Figure 10, where the sensor was embedded in a wearable mask equipped with radio-frequency identification (RFID) for continuous breath monitoring [40]. Functionalizing graphene can extend the sensing properties, and the sensors can be used for the selective detection of analytes with enhanced performance. A similar procedure was adopted for the development of multifunctional wearable sensing devices. The simultaneous monitoring of multiple physiological signals and volatile organic compounds (VOCs) was achieved via the functionalization of graphene films [22]. Here, the sensor was mounted onto the wrist to monitor the pulse and respiration rate. Respiration monitoring during sleep is also essential, as obstructive sleep apnea is one of the most common sleep disorders [99]. The printed sensor, placed on the human body, is capable of detecting breathing movements by measuring the change in magnetic vectors. 

Some other strategies were also developed to monitor respiratory rate. A colloidal dispersion of Si nanocrystals was spin-coated onto a PI substrate for the development of humidity and respiratory-rate sensors [100]. The nanocrystalline sensor showed higher sensitivity with a fivefold change and a fast recovery time (40 ms). A high response helps in the real-time monitoring of human respiration and water evaporation rate on skin. A wearable smart clothing developed through the heterogeneous integration of off-the-shelf sensors was used to independently monitor various respiratory-related signals [91]. Thermal flow sensors on biodegradable printing paper constituted another interesting idea presented recently using solvent-free low-cost graphite [92]. Using a multi-core optical fiber, together in a smart textile, was implemented for heartbeat and respiration monitoring [50]. The sensor was incorporated into a wearable textile and was sewn onto clothes for the simultaneous monitoring of both physiological activities. Two of the most prominent biomarkers (i.e., acetone and alcohol detection) in respiration are of particular interest, and different materials and strategies were implemented for the selective detection of these two analytes. The latest developments in acetone and alcohol detection human respiration are summarized in the two sub-sections below.

#### 5.2.3. Acetone for Diabetes Detection

Acetone is a particular biomarker, found alongside many other VOCs in human exhaled breath, which can be used as an early indicator for the existence of diabetes mellitus in human beings [41,101]. A high level of ketones (acetone) is produced as a result of burning fats in the human body caused by insufficient insulin production to break down glucose. The produced acetone is expelled from the human body through the lungs via breathing and partly in urine through diffusion [101]. The selective detection of acetone levels in human breath can, therefore, give indications for pre-diabetes as an early alarming sign. Conventionally, gas chromatography coupled with mass spectrometry (GC–MS) is used to determine the trace levels of VOCs in human breath; however, portable systems are desired for wearable, real-time, and continuous monitoring. The latest developments in nanoscale materials, especially the thin films of metal-oxide semiconductors (MOX), resulted in the development of miniaturized sensors that are more suitable for handheld monitoring systems [102,103]. The higher surface-to-volume ratio of nanomaterials and the porosity in the thin films were reported to be sensitive to minute trace levels (i.e., parts per billion (ppb)) of the gas analytes. Representative MOX semiconductor materials such as SnO_2_ (tin oxide), ZnO (zinc oxide), WO_3_ (tungsten oxide), CuO (copper oxide) nanowires, In_2_O_3_ (indium oxide), Fe_2_O_3_ (ferrous oxide), etc. were used for sensing various gases in human breath [22,101,102,103]. MOX gas sensors usually require a micro-hotplate to activate the redox reactions occurring at the sensing layer [104]. The higher sintering temperatures of MOX materials hinders their use on substrates with low glass-transition (T_g_) temperatures, i.e., >150 °C. Therefore, carbonaceous materials, particularly MWCNTs and their nanocomposites, fulfill the criteria of low-temperature sintering and room-temperature sensing capability [105,106]. Selectivity toward acetone can be enhanced via the functionalization of MWCNTs with acidic solutions, such as 16-mercaptohexadecanoic acid (MHDA), etc. [105]. Most recent sensors based on MWCNTs were developed on polymeric substrates, which requires heterogeneous integration onto wearable modules to enable the sensors for continuous monitoring.

#### 5.2.4. Alcohol Level Detection

**Figure 11:**
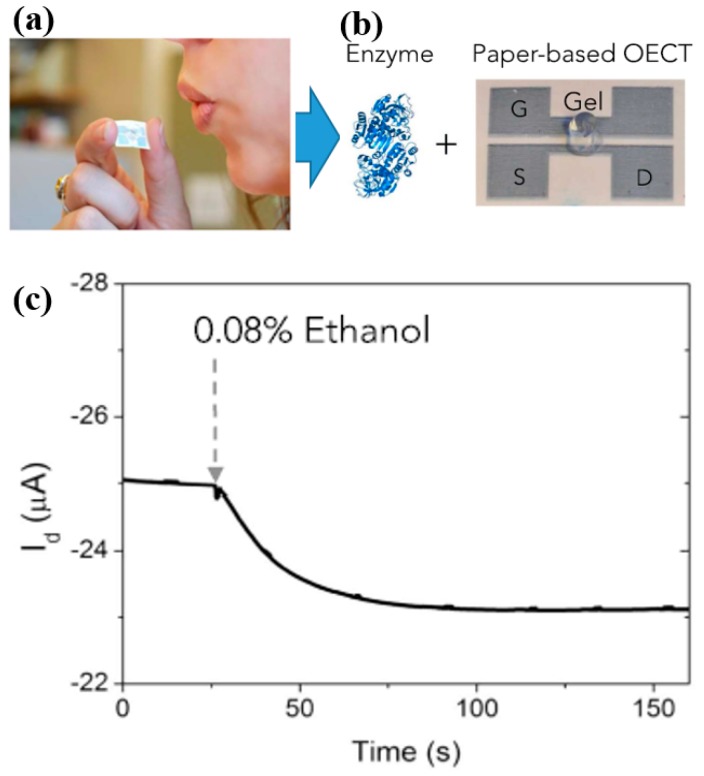
Concept of the organic electrochemical transistor (OECT) breathalyzer. (**a**) Simply breathing on the printed PEDOT–PSS OECT allows for alcohol detection. (**b**) The alcohol dehydrogenase (ADH) enzyme and the OECT are the key components of the sensor. The OECT is printed on paper, and comprises channel, source (S), drain (D), and gate (G) electrodes made of PEDOT–PSS; the enzyme electrolyte gel is deposited onto the OECT bridging the channel and gate. (**c**) I_d_ response of the OECT upon exposure to ethanol [42] (reproduced with permission).

Measuring alcohol levels in human breath is a well-known technique practiced to monitor drivers’ alertness. The conventional procedure for such analysis is to use breathalyzers for the estimation of blood alcohol content (BAC) from the concentration of ethanol in the exhaled breath. Self-assessment using wearable biosensors is considered a more effective way on the driver’s side to know if he is able to drive safely or not. This is a rather challenging task; however, researchers are still interested in exploring ways to implement such sensors, which could somehow alert the person about their ethanol content. Few researches were carried out where wearable sensors were demonstrated to monitor alcohol levels in human breath or in sweat. MOX-based nanomaterials are considered to be more responsive; Zn-based sensors (i.e., ZnO and ZnTiO_4_) in particular were explored as wearable ethanol gas sensors [107,108]. A co-planar capacitive structure was developed using ITO (indium tin oxide) electrodes and a ZnO sensing layer. The sensitivity was enhanced by irradiating the ZnO layer with ultraviolet (UV) light while testing against ethanol vapors. A ZnO-based wearable sensor was demonstrated through the quantification of an ethanol metabolite, i.e., ethyl glucuronide [109]. A low-cost and disposable breathalyzer was developed using an organic electrochemical transistor (OECT) on paper substrates, as shown in Figure 11 [42]. The device was printed using an organic conductor (i.e., PEDOT–PSS), with alcohol dehydrogenase (ADH) as its cofactor, functionalized in a gel and placed in the OECT channel for selective detection at minute levels. In the latest development, a lancet-free approach was proposed for simultaneous monitoring of alcohol and glucose in human sweat [39]. Sensors were developed by integrating ZnO thin films in a nanoporous flexible electrode system. Selectivity toward both the analytes was enhanced using alcohol oxidase and glucose oxidase enzymes, which showed a significant response at very low volumes (1–3 µL) of human sweat. There is less reported work related to wearable alcohol sensors, and the field has the potential to be explored further by developing breath- or sweat-based wearable systems. Control systems, whereby the subject is directly or remotely (by an accompanying person) monitored, need to be investigated, so as to allow the feasibility and acceptability of the system by the community.

#### 5.2.5. Hydration/Dehydration

Water comprises approximately 60% of an adult human being, which plays a significant role in maintaining the physiological health and activities of body organs. Disorders in water level in the body may lead to fatal chronic conditions, and water is, therefore, required at the desired level. Both increased and decreased levels have their own repercussions; however, a lower level is more serious, and severe dehydration may lead to different health complications. Monitoring of hydration level is important for all human beings; nonetheless, it is of higher priority for athletes doing intense exercise and equally for people (particularly laborers) working outdoors in harsh environments. Therefore, continuous monitoring of hydration or dehydration state is of particular interest and wearable sensors play a significant role in such observations.

**Figure 12:**
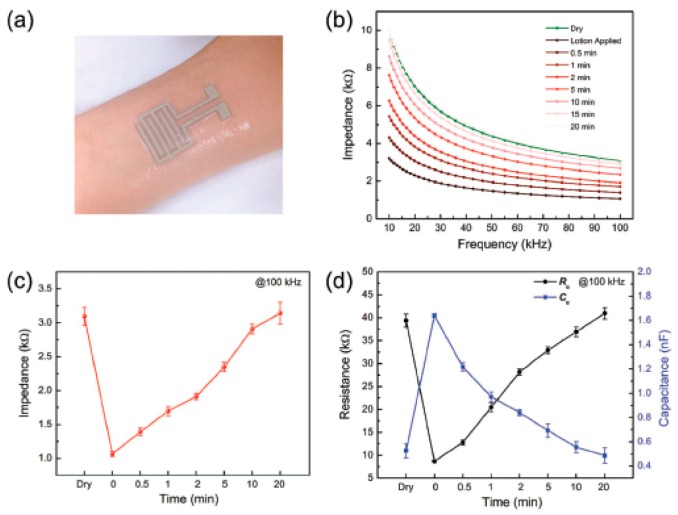
(**a**) Photograph showing the AgNW (silver nanowires) sensor placed on the inner side of the forearm; (**b**) the measured impedance change between 10 and 100 kHz from human skin before (dry) and after applying lotion; (**c**) comparison of skin impedance measured from AgNW sensor at 100 kHz before (dry) and after applying lotion; (**d**) extracted equivalent circuit model parameters (Re, Ce) before (dry) and after applying lotion [117] (reproduced with permission).

A wide variety of techniques and models were proposed for direct measuring of human body content through bioelectrical impedance-based measurements or, alternatively, through the analysis of chemical composition and concentration in sweat [16,110,111,112]. Bioelectrical impedance is the most popular and conventionally used technique, and it contains two electrodes mounted on the human body with a low-amplitude alternating current passed through them. This is a very effective way to measure the human body water content and is a common procedure for measuring dehydration. Frequent modifications are done to the bioelectric impedance model based on the placement of electrodes on the human body or based on putting conductive strips in wearable media, along with data readout through wireless communications [16]. Further improvements by embedding sensors on conformable materials, especially on textiles, are also rapidly underway, enabling the wearability of such gadgets more efficiently. Textile-based sensors for monitoring sweat were proposed for wearable and real-time analysis during exercises [44,113]. Analyzing sodium content in human sweat is considered one of the most effective biomarkers for hydration level measurements. Therefore, ion-selective electrodes were also developed in a sodium sensor belt, which can be mounted on the human body, allowing intimate contact and ease of wear [114]. Chlorine concentration was also explored as a biomarker for dehydration in human sweat, using chloride-selective electrodes [115]. Printing AgCl paste is the most common practice to develop reference electrodes on polymeric substrate using screen-printing technology. Wireless epidermis sensors mounted directly on human skin represent another interesting approach for measuring hydration and strain levels in the human skin [110,111]. Capacitive electrodes were developed, exploiting the LC (inductor capacitor) resonator concept, embedded in ultrathin and stretchable classes of materials. This system provides useful information about dehydration against the changes occurring in human skin. To simplify the sensor data readout and continuous monitoring, a RFID sensor patch was developed on a thin polymeric substrate, which can transmit data directly to a smartphone [116]. The adhesive RFID sensor patch was mounted directly on human skin for hydration monitoring, in addition to other biomarker concentrations in sweat. Recently, nanoscale materials were also exploited for developing skin-mounted hydration sensors. Nanocomposites of Ag nanowires mixed with PDMS were developed in interdigital electrode (Figure 12) structures for continuous dehydration monitoring through impedance measurements [117]. A fully packaged system was developed in the form of a wristband together with the sensors, a network analyzer chip, and a Bluetooth module, enabling the real-time monitoring of hydration levels of the human body. Graphene-based tattoos are the advanced version of wearable sensors, developed to target several biomarkers including the hydration of the human skin [118]. Graphene-based tattoos are patterned in serpentine structures through a wet-transfer dry-pattering approach. Organic materials containing a linked chain of crown ether were printed as Na^+^-binding elements for the development of a simple hydration sensor [119]. The ideal polymeric molecule containing a linked chain of crown ether is poly[(dibenzo-18-crown-6)-co-formaldehyde], which was evaluated by comparing the device characteristics with a reference device without the crown ether. Thick film-based sensors also showed promising sensing against hydration levels, in addition to other biomarkers, by exploring the electrochemical properties of the printed transducer layers. Screen printing was used to print thick-film reference electrodes with different film thicknesses to investigate the stability of potential and hydration responses [75]. Printing of hydration sensors is a seldom explored area, which has huge potential for further investigation. As most of these types of sensors are desired to be in conformal contact with the human skin, biocompatibility of all the involved materials and substrates should be of prime focus.

#### 5.2.6. Temperature

**Figure 13:**
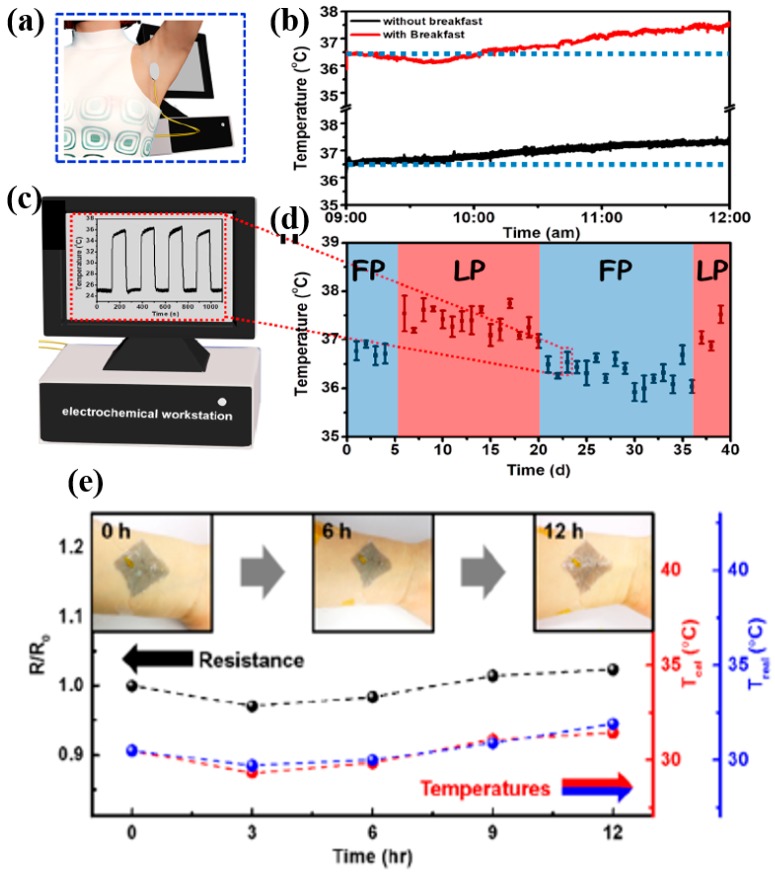
(**a**) Schematic of skin-mounted temperature sensor; (**b**) temperature curves with and without breakfast; (**c**) on–off cycles of the thermal response between in vitro body temperature and room temperature; (**d**) real-time monitoring of in vitro body temperature of a volunteer without breakfast (black) or with breakfast (red). Temperature measurement was of a female volunteer at 5 pm every day. The follicular phase (FP) and luteal phase (LP) tested according to the temperature variation are clarified by blue and red regions [46] (reproduced with permission). (**e**) Changes in the relative resistance (left *y*-axis), measured every 3h during a 12-h attachment onto an arm. The red and blue data correspond to the temperatures estimated from the relative resistance change of our device and that measured by an infrared (IR) thermometer (right *y*-axis) [131] (reproduced with permission).

Wearable temperature sensors are widely investigated, exploring a large variety of sensing materials and printing technologies. Temperature sensors in wearable applications are considered for two purposes, i.e., for continuous human-body temperature and ambient temperature measurements. For human-body temperatures, sensors are mounted directly on the skin epidermis or kept in intimate contact with the skin while using a separable entity in a wearable gadget [120]. Continuous monitoring of human-body temperature is of particular interest, especially for patients with prolonged chronic diseases, ordinary sickness, unconscious or injured individuals under anesthesia and surgical operations, and, last but not least, laborers working outside in harsh conditions. Wearable temperature sensors are also interesting for electronic skin applications and are widely explored in addition to many other sensing devices, aimed at the advancement of industrial and particularly social robots [121,122]. Printed thermal sensors are mostly based on the resistance measurements of a metallic structure against temperature rise, and the thermal coefficient of resistance (TCR) values are used to determine the temperature response accordingly [123]. Figure 13 shows representative examples of human-body-mounted temperature sensors providing data in real time.

A large variety of thermal sensors were developed in the last couple of years using intrinsic conducting and nanocomposite materials in different geometrical shapes to enable wearability at minimum bending angles [124]. An all-printing approach makes the fabrication of temperature sensors simple and cost-effective, allowing the development of single layers of patterned conducting lines in various shapes (meander, spiral, or circular). One such sensing device was developed using a polymeric blend with SWCNTs for the simultaneous detection of temperature and CO_2_ gas [125]. The mounting of sensors directly onto the epidermis requires substrates with properties similar to the human skin, such as biocompatibility, air/oxygen permeability, being waterproof, etc. Meeting these demands in the current scenario is quite challenging. One such development was reported, presenting breathable and stretchable temperature sensors inspired by human skin [126]. Sensors were also developed via the transfer printing of Cu strips onto semipermeable polyurethane films. To avoid the photolithography stages, an inkjet-printed graphene/PEDOT–PSS temperature sensor was developed on a skin-conformable polyurethane substrate [127]. Skin-mounted biosensors for the simultaneous detection of sweat metabolites, electrolytes, and temperature were developed in a single patch for prolonged wearability during exercise [61]. The multiplexed sensors were fully integrated onto a polymeric substrate, enabling precise wearability, and they were functionalized to enhance selectivity toward target analytes. Another similar approach to the simultaneous monitoring of sweat pH and skin temperature was proposed by measuring through an ion-selective field-effect transistor (ISFET) and an integrated temperature sensor, respectively [1]. Ag was printed for the interconnection electrodes, while PEDOT–PSS was printed for the temperature-sensing layer. The sensors were tested in real time by attaching the sensor patch on the neck of the test subject while doing exercise.

In the latest developments, the printing and performance of different nanoscale materials were also investigated. Resistance changes against the temperature rise of sensing layers connected by an interconnecting conductive paste were monitored. A comparative study of carbonaceous materials (i.e., rGO, SWCNTs, and MWCNTs) was performed for temperature sensing based on the responsivity and stability for prolonged use [128]. It was found that rGO presented promising stable results in various environmental conditions (humidity, pressure, and test gases). The sensors were responsive despite being insulated with an overlying layer as protection against all these environmental variations. Graphene nano-wells show tremendous temperature coefficients of resistance (i.e., 180% K^−1^), which is an interesting development for the detection of human body temperature [129]. A biocompatible conductive green electrolyte is another attractive candidate for in vivo and in vitro body temperature measurements [46]. The electrolyte is developed using complexes of aliphatic diols and calcium chloride (CaCl_2_), and the resistance change of the conductor is measured against the temperature rise. A customizable sensor developed through 3D printing is capable of monitoring the temperature and pressure as step sensors, and data are read out visually using a human skin-like colored substrate [130]. A multilayer stack of pressure and temperature sensors was printed and integrated as a single patch using a 3D integration approach. An interesting technique to allow the self-adhesion onto human skin, inspired by an octopus-mimicking adhesive, was developed using structures of PDMS substrate [131]. A resistance-based temperature sensor based on a nanocomposite of a poly(*N*-isopropylacrylamide) (pNIPAM) hydrogel, PEDOT–PSS, and CNTs was developed, and it exhibited a good thermal response of 2.6% °C^−1^ at 25–40 °C in the range of human body temperatures. A very highly stretchable and self-healing hydrogel based on a polyacrylamide/carrageenan double network (DN) was also exploited as a thermistor [132]. The higher stretchability (i.e., 0–330% strain with a sensitivity as high as 2.6% °C^−1^ at higher strain) enables these types of sensors to be used on body joints or on very irregular surfaces, analogous to the self-healing capability of human skin. The monitoring of active heating, possibly required for applications such as heat therapy, perioperative warning, controlled transdermal drug delivery, etc., is another interesting feature, whereby integrated temperature sensors are used for accurate control [133]. Devices for a similar approach were implemented using a stretchable aluminum heater and gold-based RTDs (resistance temperature detectors) for temperature feedback control [133]. This tattoo-like heater stretchable and wearable patch was developed on a soft medical tape using a cut-and-paste approach. The patch can be mounted on any part of the body and follows the skin deformation during flexures without any significant restraints. All these developments and innovative strategies targeting a variety of application areas for wearable temperature sensors show the extreme interest of the researcher community at large.

#### 5.2.7. Motion/Activity Monitoring

**Figure 14:**
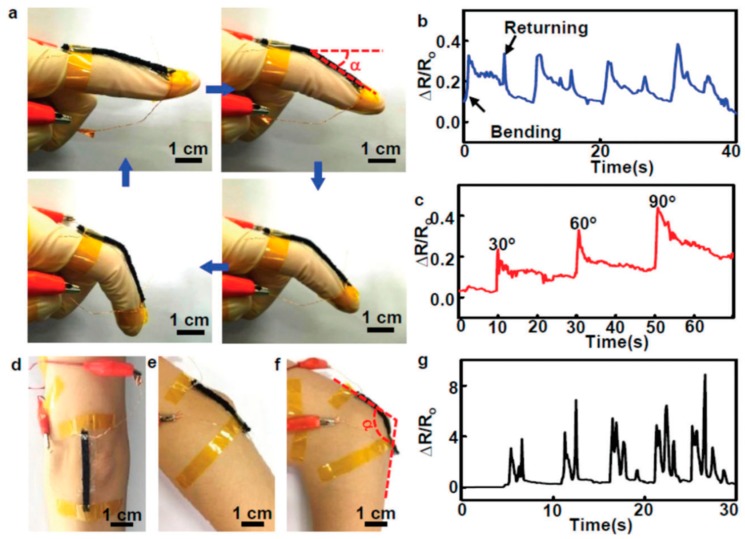
Detection of large-strain human motions with the strain sensor attached to the human finger and elbow joints. (**a**) Photographs showing the bending test of the finger; (**b**) the corresponding relative resistance change of the sensor in response to the finger bending; (**c**) the corresponding relative resistance change of the sensor in response to the different finger-bending angles; (**d**–**f**) photographs showing the bending test of the elbow; (**g**) the corresponding relative resistance change of the sensor in response to the elbow bending [137] (reproduced with permission).

Motion or activity monitoring of moving objects is a widely explored area in the field of wearable electronics. Wearable motion detection is mostly based on strain sensors, whereby the change in the base resistance is considered as a recognition for motion-related activities [134,135]. Motion detection is of particular interest for prosthetic limbs, soft robots, and physically impaired or elderly persons requiring continuous activity monitoring remotely [136,137]. State-of-the-art sensors and systems come with major hindrances such as bulkiness, rigidness, non-wearability, heavy weight, etc. which make it challenging to carry around the system for continuous monitoring [136,137]. Therefore, a lot of research is devoted to the development of lightweight wearable systems based on thin-film electronics and sensors directly printed on lightweight conformable polymeric or textile-based substrates [138]. The wearable suits are sometimes termed electronic skins, equipped with a few sensors to replicate the sensing capabilities of human skin [139,140]. Different approaches were pursued to develop these sensors, for instance, using discrete sensors at different parts of the body or through a well-connected wearable suit [136,141]. The soft motion-sensing suit contains sensors suitably placed at the joints that are involved in moving back and forth, triggering the sensing device. Human gait detection was presented, whereby strain sensors were embedded in a wearable suit to monitor the motion activities [142]. Strain sensors were developed using liquid metal embedded into an elastomer and placed onto the hip, knee, and ankle joints to monitor their bending angles, as shown in Figure 14.

Nanoscale materials play a significant role in developing motion detection sensors in their pristine state or in composites by mixing them with elastomeric polymers. Embedding conductive fillers in rubber-based materials is also advantageous due to their higher stretchability and compliant integration onto non-planar surfaces. One such approach was reported, whereby a natural rubber was used as a base for infusion of liquid-exfoliated graphene for developing a conductive composite [143]. The change in resistance recorded for these structures was reported to be a 10^4^-fold increase with a maximum strain of 800%. This makes the sensor highly sensitive, and the dynamic response enables it to be mounted onto various body parts, especially joints, for continuous motion monitoring. Silver nanowires were printed using Ecoplex as a dielectric layer to develop capacitive sensors for wearable multifunctional sensors [144]. The sensors were used to detect strain, pressure, temperature, and touch of various human body parts in various physiological conditions. A unique double-helical CNT array was used to develop a strain sensor to detect hand motion [145]. The array matrix was capable of measuring strain up to 410% with low hysteresis and high sensitivity to slight motion. Nanocomposites of CNT and Ecoplex resulted in ultra-stretchable and skin-mountable strain sensors for motion detection [146]. The percolation network of CNTs provides conductive tracks, while Ecoflex provides a stretching medium to enable deformations caused at the joints or muscles during body motion. The change in resistance in the bulk resistance of the percolation layer was exploited as a measure relative to the degree of expansion. In another approach, the 3D printing of CNT and polyurethane nanocomposites was reported, whereby thin filaments were extruded to form strain sensors [147]. Similarly, CNT/PDMS nanocomposites also showed very promising results for wearable strain-based motion sensors [49,137,138]. The 3D printing capability enables various mixing ratios and viscosities of CNT/polyurethane for reaching percolation mixes ideal for strain-sensing applications. A multifunctional sensing device was printed on conformable substrate, utilizing Ag, CNTs, PEDOT–PSS, and ZnO for the detection of various signals related to real-time human health monitoring [34]. Sensor patches are designed with two components, i.e., disposable and nondisposable components. The disposable part can be mounted onto the skin and contains printed sensors to monitor temperature, acceleration, electrocardiograms, and kirigami structures, allowing skin stretching. In recent developments, a novel composite material was developed by making a conductive sponge, impregnating carbon black with a shear-thickening gel and polyurethane [134]. The developed sensor provided a reliable safeguarding performance by reducing the impacting force by 44%, allowing the simultaneous detection of human body motion. Liquid-metal and conductive iono-elastomer-based strain sensors attracted significant interest recently, and they were used to detect large deformations with higher sensitivity [135,148].

#### 5.2.8. Pressure and Strain

Pressure and strain sensors are among the key wearable systems and electronic skin applications to determine soft touch, grasp and handling/manipulation, the classification of material surfaces, the monitoring of human pulse rate and motion activities, etc. [139,149]. Various sensors and systems based on pressure and strain sensors were described in some of the previous sections, using them as complementary devices to establish evaluations based on the data acquired from these sensors. However, using pressure or strain sensors in wearable electronics has its own importance. Therefore, the field is widely explored, utilizing various materials and techniques in different geometric structures to link the resulting transduction to the level of pressure or strain applied on the subject. More interest can be found in using materials in their pristine and engineered forms (especially nanocomposites), processed from their solutions through printing technologies [6,150]. The sensors are usually supported by additive structures such as pyramids or elevated squares to enhance the localized pressure. These pressure–concentration structures are ideal for an array of sensors which reduces the cross-talk between neighboring units and also compensates for a loose touch or compressibility. One such approach was reported by developing a highly stretchable resistive pressure sensor using a PDMS-based resistive pressure sensor supported by a micropyramid array [151]. A conductive electrode was grafted onto the micropyramid here against the conventional percolation mechanism, where a slight deformation (both by compression and stretching) was sensed through the enhancement caused by these pyramid structures.

**Figure 15:**
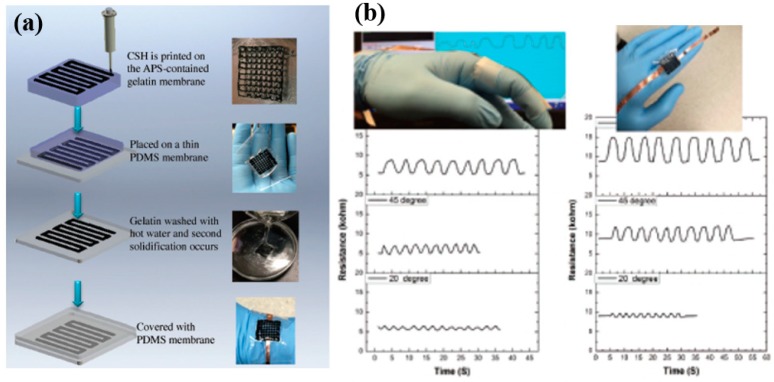
Three-dimensional (3D) printing characterization, preparation, and application. (**a**) Preparation of a 3D-printed wearable sensor with the CSH hydrogel. A real-time bodily motion monitoring system using smartphones and a 3D-printed CSH wearable and flexible sensor; (**b**) resistance variation of the CSH hydrogel strip (left) and 3D-printed sensor (right) attached onto the index finger, as the finger was subjected to repeated bending and relaxing from 0° to 20°, 45°, and 90° [155] (reproduced with permission).

The incorporation of nanomaterials into the elastomeric matrix or into a sandwiched structure plays a significant role in developing pressure and strain sensors [6,144]. Conductive networks are established between the aggregates of conductive fillers and they provide a base resistance when no deformation forces are applied. In addition to the physical contact of the conductive fillers, tunneling between conductive fillers can also contribute to base resistance. The base resistance changes with respect to the applied pressure or stretching force. Ultrathin gold nanowire-based pressure sensor sheets were developed using a paper substrate [81]. Au nanowires were impregnated in a tissue paper and were sandwiched between two thin PDMS sheets to enable the stretchability of the structure. A simple drop-coating method was employed for the impregnation process, which was low-cost and scalable for large-area deposition for mapping spatial pressure distribution. The developed sensors were highly sensitive and were applied for the pressure of human pulse. A similar approach was presented by preparing a carbon paper using a tissue paper through a high-temperature pyrolysis process [15,152]. The prepared carbon paper was mixed with PDMS to make a strain sensor which was applied for breath monitoring and robot controlling. However, the integration of multiple layers was sometimes time-consuming, prone to detachability and breaking; therefore, a more robust and direct integration of sensitive materials onto the desired wearable gadget is required. For this purpose, an embedded 3D printing approach for developing strain sensors directly onto a highly stretchable elastomer was reported, as shown in Figure 15 [153]. A viscoelastic ink was extruded through a deposition nozzle onto the target substrate reservoir. After printing, the reservoir and filler fluid were co-cured to make a monolithic layer. The localized deposition through printing technologies enabled the development of highly sensitive and arrayed sensors in various configurations. A pressure sensor was printed in the form of a transistor using 3D self-organized organic semiconductor microstructures [154]. Due to its higher sensitivity, the sensor was applied for real-time monitoring of radial-artery pulse pressure, as well as for touch sensing in the electronic skin of a prosthetic hand.

Some new innovations were introduced in the field lately by developing new types of materials and structures. Conductive self-healing hydrogels attracted interest, as they mimic the human skin and could possibly be exploited for a wide variety of sensors and wearable electronic applications. These hydrogels were recently applied for the development of pressure and strain sensors [155]. The 3D printability of these hydrogels enables their development on a range of geometrical structures complying with the desired targeted shapes. Microstructuring of the sensing layer to compensate for the deformations and retain the initial electrical conductance after relaxation is also of particular interest. In this context, a wearable pressure sensor was developed using a nanocomposite of CNT/PDMS arrays based on ultraviolet/ozone microstructuring [12,150]. The microstructuring technique was controllable, cost-effective, and highly efficient as it was conducted at room temperature in ambient conditions. An active matrix of large-area pressure sensors was produced using carbon nanotubes on flexible substrates, targeting an electronic skin application [10]. An active matrix of 16 × 16 transistors was made from highly purified CNTs, exhibiting higher mobility and current density. The sensors operated at small voltages (i.e., 3 V) and were reported to show a faster response than human skin (<30 ms). The sensor was aimed at an electronic skin application in soft robots, as well as for prosthetic solutions. The area of pressure and strain sensors is widely explored and was applied to develop multiple wearable health and physiological monitoring applications.

#### 5.2.9. Gas Sensors

**Figure 16:**
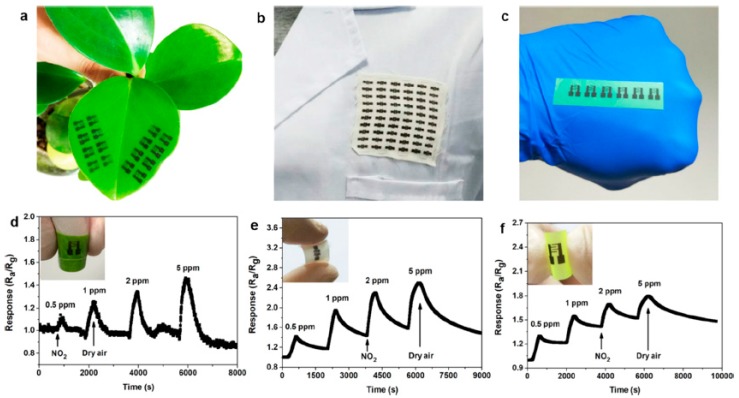
Photographs of gas sensor arrays prepared on (**a**) living plant leaf, (**b**) silk stitched onto a lab coat, and (**c**) portable sticker attached onto the human body. Dynamic responses are shown for the samples fabricated on (**d**) leaf, (**e**) silk, and (**f**) portable sticker [168] (reproduced with permission).

Gas sensors witnessed tremendous research interest in the wake of the latest advancements in nanoscale materials. The solution processability of a wide range of nanomaterials and their integration into polymeric substrates enabled the development of flexible and conformable gas sensors. Gas sensors are categorized into different types; however, the detection of toxic gases in the surrounding environment and the monitoring of exhaled gases in human breath are of particular interest and are, thus, considered in this review. In this scenario, toxic gas sensors are ideally deployed onto wearable gadgets placed at any position on the human body exposed to the outside atmosphere for gas detection. In the second case, the sensing gadget is required to be directly in the pathway of exhaled breath to detect the target gas analyte. Gas sensors used for human breath analysis are described in detail in previous sub-sections focused particularly on acetone detection for diabetes. Various other sensors were also considered to selectively detect other volatile organic compounds, which are considered as biomarkers for specific diseases in the human body. This sub-section mainly focuses on wearable gas sensors (other than for exhaled breath) deployed on a conformable substrate for continuous monitoring. Figure 16 shows an interesting overview and the prospective scope of printed and wearable gas sensors developed on a variety of unconventional substrates.

Gas sensors are sometimes termed electronic noses, used as sniffers in various applications [156,157]. A CNT/polymer composite was inkjet-printed to develop an electronic nose for armpit odor analysis [156]. The sensor was capable of classification of different armpit odors released from the human body and classified the level of skin hygiene against different physiological activities. Ammonia detection is also one of the main biomarkers in human health monitoring, and selective detection helps in the early diagnosis of several diseases, such as kidney or liver failure. MOX semiconductor-based gas sensors are ideal for the detection of toxic gases, as well as volatile organic compounds [30,158,159]. MOX-based sensors are discussed in the section on acetone detection for diabetes analysis. ZnO-based light-controllable wearable gas sensors for ethanol detection were reported [107]. ITO electrodes were used to connect a thin film of ZnO nanoparticles to a PET substrate. The sensor gave higher response under UV irradiation, in addition to its excellent photoresponse. Gas sensing in the form of transistors was recently reported for enhanced sensitivity using an MOX compound, i.e., amorphous indium–gallium–zinc oxide (a-IGZO) [160]. Carbonaceous materials (i.e., graphene and CNTs) play a significant role in developing gas sensors at low temperatures [161,162]. A single yarn of a graphene-based ultrasensitive and highly selective sensor showed remarkable performance for a wearable gas sensing application [163]. This unique sensor was mounted on an e-textile, which possessed chemical inertness to several detergents while washing and was highly stable under mechanical bending for more than 1000 bending cycles. Reduced graphene oxide was used on yarn and molecular glue through an electrostatic self-assembly. The sensor was highly responsive toward NO_2_, as well as acetone, ethanol, and CO_2_. Functionalized graphene films were used to develop multifunctional wearable sensing devices [22]. Functionalization enhances the selectivity and sensitivity for simultaneous monitoring of physiological signals and volatile organic compounds at the same time. An inkjet-printed ammonia gas sensor was developed using a nanocomposite of PEDOT–PSS/graphene, which showed a higher response [164]. An interesting feature in this development was the use of a normal office printer (HP deskjet 2000) instead of using conventional inkjet systems.

Engineered materials were also explored recently for developing highly sensitive and selective gas sensors, matching the processing conditions of flexible substrates. For instance, transfer printing of AlGaN/GaN-based (aluminum gallium nitride/gallium nitride) gas sensors was developed on plastic substrates [165]. The sensors were developed on the host h-BN substrate and were subsequently transferred to polymeric substrates deterministically without any degradation in the performance. Furthermore, a novel approach was presented for enhancing the sensitivity of gas sensors by converting inkjet-printed Ag electrodes into porous Au counterparts [166]. Ag-based IDEs were printed via inkjet to make a chemiresistive gas sensor using SWCNTs as a sensing layer. A two-step wet chemical process was adopted to convert Ag IDEs into porous Au in ambient conditions, while maintaining the adhesion to substrate. The proposed concept was applied for detect diethyl ethylphosphonate (DEEP, a simulant of the nerve agent sarin), showing a fivefold higher response than similar conventional sensors. Quantum dots were explored lately to develop the first ever fully stretchable and humidity-resistant gas sensors [167]. A crumpled quantum-dot sensing layer was created using graphene as an electrode on an elastomeric substrate. An enhanced sensitivity toward NO_2_ was achieved with a fivefold improvement in the sensor response at room temperature. Sensors were mounted on human fingers at the joints for analysis of the sensor under various stressed and relaxed conditions, while performing gas-sensing tests. A large-area gas sensor array was developed through spray deposition utilizing a hybrid nanostructure of AgNPs with carbon [168]. The proposed structure included SWCNTs as electrodes, whereas AgNP-decorated reduced graphene was exploited for the selective detection of NO_2_ at 6–20 ppm of concentration. A practical demonstration of mounting the sensors on a human arm was presented with no major deviations in the sensor response when compared to the data in a planar configuration. All the representative examples mentioned and the focal research interest in developing gas sensors on polymeric and conformable substrates show the importance of these sensors for the fast-growing real-time wearable health-monitoring systems. Table 2 summarizes materials, potential substrates, sensing mechanisms, and fabrication procedures for biosensing devices based on physiological activities.

## 6. Conclusions and Future Perspectives

Significant progress was witnessed in the field of wearable sensors and systems. Continuous monitoring of human health through non-invasive approaches is not only restricted to patients suffering from chronic diseases, but also has widespread use in fitness, entertainment, the fashion industry, etc. The recent advancements in solution-based nanomaterials and their processing through printing technologies enabled the cost-effective manufacturing of diverse biosensors on a wide variety of flexible substrates. The great surge in wearable sensors for the recognition of biomarkers in skin-excreted biofluids, as well in the continuous monitoring of physiological activities, shows a promising future in biomedical applications. 

Wearable sensors and systems are foreseen to revolutionize the field of medical diagnostics; however, these developments are still confronted by several challenges. The challenges are diverse and range from manufacturing to materials, substrates, selectivity, convenient signal readout circuitry, multi-functionality, simultaneous monitoring, and adoptability of these sensor patches by humans. The integration of advanced nanoscale materials on polymeric substrates is the key to enabling the development of conformable electronics. The lower glass-transition temperature of polymeric substrates is a major obstacle to the development of densely integrated devices with ultrathin sensing films of inorganic semiconductors. Therefore, solution-based additive manufacturing techniques are used for sensor development resulting in macro-scale devices at lower density. This further leads to challenges arising from integrating different materials in multi-layered device structures that require different manufacturing processes. The biocompatibility of materials and substrates is another major concern for wearable electronics, especially for sensors mounted on the skin/epidermis. Physical, mechanical, and chemical properties of materials and substrates need to be matching to avoid thermal, electrical, and multi-layer integration mismatches. Bioresorbable or self-destroyed materials and substrates are potentially needed to be developed for implantable electronics.

Operational challenges of wearable electronics represent another paradigm, where special conditions and pretreatments of the sensors are desired to enhance the sensitivity, selectivity, stability, limit of detection, etc. Some of these treatments such as localized heating using microheaters in gas sensors, or chemical treatments to restore the initial stable values cannot be applied while sensors are installed on wearable gadgets. The simultaneous monitoring of multiple analytes is also challenging as cross-talk between different sensors influences their selective detection. Increasing the density of sensors, data processing units, and wireless communication channels would require more power and is, therefore, challenging when trying to maintain the same level of operation for long durations. In this scenario, long-lasting wearable batteries, supercapacitors, efficient solar and fuel cells, etc. are highly desired to enable the smooth and optimal operation of the whole system. The development of compact and highly efficient communication tools/channels and protocols is also needed, enabling the smooth transmission of data between sensor nodes and the computing device. Data security is another big concern for wearable electronic systems, as they involve a huge amount of personal information of the wearer. Security breaches through cyber-attacks or any other mishandling activity may result in the wrong interpretation of personal health status, which could lead to severe repercussions. An inclusive research strategy is, therefore, needed to be designed to tackle the challenges of this cross-disciplinary field, and active collaborative research will play a significant role in the commercial success of these new types of sensors.

## Figures and Tables

**Table 1:** Summary of representative materials, substrates, mechanisms, and fabrication procedures for fluidic-based sensors.

Sensor Type	Materials	Substrates	Mechanism	Fabrication	References
**Glucose Sensors**	Ag/AgCl	Polyurethane	Iontophoresis (IP)	Screen Printing	[54]
	Cr/Au, Cr/Pt	PI/PDMS	Electrochemical	Transfer Printing	[48]
	In_2_O_3_, Au	PET	Transconductance by using FET	Shadow Masking	[24]
	Ag/AgCl	PET	Chronoamperometric	Lithography	[58]
	nPt	Flexible stainless steel	Electrochmical	Electroplating	[55]
	Ag/AgCl, PEDOT-PSS	PET	Amperometric	Vacuum Deposition	[15]
	Graphene Oxide, Au, Pt	PI	Amperometric	E-Beam Evaporation	[21]
**Lactate Sensors**	Carbon, Ag/AgCl	Textile (cotton)	Amperometric	Screen Printing	[13]
	Carbon, Ag/AgCl	Polymeric tubes	Amperometric	Manual Printing	[36]
	Graphene Oxide, Ag/AgCl		Electrochemical Chronoamperometric	Screen Printing	[63]
	MWCNTs, Ag/AgCl	Paper (Papilio)	Electrochemical	Screen Printing	[60]
	Ag/AgCl	PET	Chronoamperometric	Lithography	[58]
	Carbon, Ag/AgCl	PET	Electrochemical	Screen Printing	[27]
	3-Aminophenylboronic acid (3-APBA)	PET	Impedimetric	Screen Printing	[64]
**pH Sensors**	Au, Polyaniline, CNT	PDMS	Electrochemical	Layer by layer (LBL)	[9]
	CNT, Ag	PI	Conductometric	Aerosol Jet Printing	[68]
	SWCTs	Liquid Crystal Polymer	Conductometric	Inkjet, Screen Print	[72]
	Ag/AgCl, KCl, RuO_2_	Glass	Electrochemical	Screen Printing	[73]
	TiO_2_	Alumina	Electrochemical	Screen Printing	[67]
	PVB-Ag/AgCl, PANI,	PET	Electrochemical	Electrochemical deposition	[69]

**Table 2:** Summary of representative materials, substrates, mechanisms, and fabrication procedures.

Sensor Type	Materials	Substrates	Mechanism	Fabrication	References
**Pulse Rate**	Graphene Oxide	PET, PI	Conductometric	Transfer Printing	[22]
	PEDOT-PSS, PVDF-TrFE	PEN	Piezoelectric	Screen Printing	[23]
	PDMS, eGaIn	PDMS (tube)	Conductometric	Injection	[85]
	PVDF-TrFE, Al, Ag	PI	Piezoelectric	LBL	[82]
	Au	PDMS	Piezoresistance	Impregnation & Sandwiching	[79]
	Graphene Oxide, Au	Fabric (Facemask)	Humidity (variations)	Drop Casting	[40]
**Respiratory/Breath**	Graphite, SiO_2_	Cellulose Acetate	Humidity	Hand-painting	[38]
	Graphite	Paper	Conductometric	Hand-painting	[90]
	Silicon-nanocrystal	PI	Humidity	Spin coating	[98]
	CNTs	PDMS	Strain	Laser Scribing	[94]
	PVDF-TrFE	PDMS	Piezoelectric	Molding	[96]
	ZnO, Au	PI (nanoporous)	Impedance	E-beam, Sputtering	[39]
**Alcohol/Acetone**	ZnO, TiO_2_, Cu	Alumina	Chemiresistive	Screen Printing	[106]
	Au, ZnO	PI	Chemiresistive	E-Bean, Sputtering	[107]
	PEDOT-PSS	Paper	Transconductance	Inkjet Printing	[42]
	ITO, ZnO	PET	Chemiresistive	Drop Casting, Laser ablation	[105]
	ITO, P3HT, PVP	Glass	Chemiresistive	Drop Casting	[118]
**Hydration**	Graphene, Ag/AgCl	PMMA	Impedance	Wet Transfer, Dry Patterning	[117]
	Ag, PDMS	PDMS	Impedance	Drop Casting	[116]
	Ag/AgCl	PET	Electrochemical	Screen Printing	[114]
	PANI, PEDOT-PSS, CNTs	PDMS	Resistive	Spin coating, Molding	[131]
**Temperature**	CaCl_2_, Aliphatic Diols	PLA	Conductometric	Injection	[129]
	Graphene, Ag, PDMS	PET	Resistive	Transfer Printing	[128]
	Graphene/PEDOT-PSS	Polyurethane	Resistive	Inkjet Printing	[126]
**Motion and Activity Monitoring**	Pluronic F127, EAN	Ion-gel elastomer film	Resistive	Molding	[135]
	Galinstan, mass ratio: Ga/In/Sn = 68.5:21.5:10%	Silicone	Inductive	3D-Printing	[148]
	Carbon black	Polyurethane	Strain, Conductivity	Drop and dry	[134]
	Carbon, Ag	PDMS, Cotton	Strain	Impregnation, Casting	[138]
	MWCNT, Cu	PDMS	Strain	Casting	[137]
	MWCNTs,	Polyurethane	Piezoresistive	3D Printing	[147]
**Pressure & Strain**	MWCNTs, Al_2_O_3_	PI	Pressure/TFTs	ALD, Vacuum Deposition	[10]
	Cu, CNTs	PDMS	Piezoresistive	Casting	[12]
	Conductive self-healing hydrogel	PDMS	Piezoresistive	3D Printing	[155]
	SWCNT/paper, Au, PDMS	PI	Piezoresistive	E-beam evaporation	[15]
	ITO, diF-TESADT	PET	Pressure/OFETs	3D Printing	[154]
	Carbon	PDMS	Strain	Coating	[152]
**Gas Sensors**	AgNPs, Carbon, CNT	Silk	Chemirsistive	Spray & Drop Coating	[168]
	a-IGZO	PI	Output current (TFTs)	ALD, RF-Sputtering	[160]
	Reduced Graphene Oxide	PET	Chemirsistive	Drop casting, Spin coating	[22]
	Graphene, PbS Quantum Dots,	VHB acrylic 4910	Chemirsistive	Transfer Printing	[167]
	Ag, Au	PI	Chemirsistive	Inkjet Printing	[166]
